# Strain-level bacterial typing directly from patient samples using optical DNA mapping

**DOI:** 10.1038/s43856-023-00259-z

**Published:** 2023-02-23

**Authors:** My Nyblom, Anna Johnning, Karolin Frykholm, Marie Wrande, Vilhelm Müller, Gaurav Goyal, Miriam Robertsson, Albertas Dvirnas, Tsegaye Sewunet, Sriram KK, Tobias Ambjörnsson, Christian G. Giske, Linus Sandegren, Erik Kristiansson, Fredrik Westerlund

**Affiliations:** 1 Department of Life Sciences, Chalmers University of Technology, Gothenburg, 412 96 Sweden; 2 Department of Mathematical Sciences, Chalmers University of Technology & University of Gothenburg, Gothenburg, 412 96 Sweden; 3 Department of Systems and Data Analysis, Fraunhofer-Chalmers Centre, Gothenburg, 412 88 Sweden; 4 Centre for Antibiotic Resistance Research in Gothenburg (CARe), Gothenburg, 405 30 Sweden; 5 Department of Medical Biochemistry and Microbiology, Uppsala University, Uppsala, 751 23 Sweden; 6 Department of Astronomy and Theoretical Physics, Lund University, Lund, 223 62 Sweden; 7 Department of Laboratory Medicine, Karolinska Institutet, Stockholm, 141 86 Sweden; 8 Department of Clinical Microbiology, Karolinska University Hospital, Stockholm, 171 76 Sweden

**Keywords:** Bacteria, Diagnostic markers, Bacterial infection

## Abstract

**Background:**

Identification of pathogens is crucial to efficiently treat and prevent bacterial infections. However, existing diagnostic techniques are slow or have a too low resolution for well-informed clinical decisions.

**Methods:**

In this study, we have developed an optical DNA mapping-based method for strain-level bacterial typing and simultaneous plasmid characterisation. For the typing, different taxonomical resolutions were examined and cultivated pure *Escherichia coli* and *Klebsiella pneumoniae* samples were used for parameter optimization. Finally, the method was applied to mixed bacterial samples and uncultured urine samples from patients with urinary tract infections.

**Results:**

We demonstrate that optical DNA mapping of single DNA molecules can identify *Escherichia coli* and *Klebsiella pneumoniae* at the strain level directly from patient samples. At a taxonomic resolution corresponding to *E. coli* sequence type 131 and *K. pneumoniae* clonal complex 258 forming distinct groups, the average true positive prediction rates are 94% and 89%, respectively. The single-molecule aspect of the method enables us to identify multiple *E. coli* strains in polymicrobial samples. Furthermore, by targeting plasmid-borne antibiotic resistance genes with Cas9 restriction, we simultaneously identify the strain or subtype and characterize the corresponding plasmids.

**Conclusion:**

The optical DNA mapping method is accurate and directly applicable to polymicrobial and clinical samples without cultivation. Hence, it has the potential to rapidly provide comprehensive diagnostics information, thereby optimizing early antibiotic treatment and opening up for future precision medicine management.

## Plain Language Summary

For bacterial infections, it is important to rapidly and accurately identify and characterize the type of bacteria involved so that optimal antibiotic treatment can be given quickly to the patient. However, current diagnostic methods are sometimes slow and cannot be used for mixtures of bacteria. We have, therefore, developed a method to identify bacteria directly from patient samples. The method was tested on two common species of disease-causing bacteria – *Escherichia coli* and *Klebsiella pneumoniae* – and it could correctly identify the bacterial strain or subtype in both urine samples and mixtures. Hence, the method has the potential to provide fast diagnostic information for choosing the most suited antibiotic, thereby reducing the risk of death and suffering.

## Abstract (web summary)

Nyblom, Johnning et al. develop an optical DNA mapping approach for bacterial strain typing of patient samples. They demonstrate rapid identification of clinically relevant E. coli and K. pneumoniae strains, without the need for cultivation.

## Introduction

Bacterial infections cause millions of deaths every year^1,2^, and the number is expected to rise due to the increase in antibiotic resistance, making treatment more challenging. Especially troublesome are Gram-negative bacilli encoding plasmid-borne antibiotic resistance genes, some of which are on the WHO antimicrobial resistance priority pathogen list^3^. The need for novel diagnostic techniques that can rapidly guide effective treatment is, therefore, urgent. An integral part of diagnosing infections is typing of bacterial pathogens to determine the infecting species. However, with a growing number of globally widespread particularly virulent and antibiotic-resistant variants, identification at the strain level becomes increasingly important^4^. Routinely used methods in hospitals, such as phenotypic tests and mass spectrometry (MALDI-TOF), typically distinguish infecting bacteria on a species level, and together with more high-resolution methods like multi-locus sequence typing (MLST) and whole-genome sequencing, they all require one or two overnight cultivations of the bacteria to obtain pure samples and a sufficient amount of DNA or protein for analysis^4–6^. In addition, some bacteria cannot easily be cultured in laboratories. The time to reach a complete resistance profile and hence a correct diagnosis is, therefore, limited by the growth rate of the bacterium and typically takes at least 24 h^7,8^. Also, these methods are optimized to analyse single bacterial isolates and struggle with polymicrobial samples, which are common for many types of infections. There is thus a demand for novel strategies that can rapidly identify causative bacterial pathogens from any uncultured sample, including those containing a mixture of microorganisms.

Optical DNA mapping (ODM) is an umbrella term for methods that visualize sequence-specific patterns along single DNA molecules (~50 kb or longer)^9^. We have developed an ODM assay based on the non-covalent competitive binding of the fluorescent YOYO-1 and the AT-specific, non-fluorescent netropsin to DNA that results in a sequence-specific emission intensity variation along the DNA molecule^10^. The emission intensity variation can be visualized using fluorescence microscopy whilst the DNA is stretched in nanofluidic channels^11^. Since the method relies solely on the interaction of YOYO-1 and netropsin with DNA, it is agnostic to the DNA in the sample, which means that any bacterium can be investigated without, for example, designing primers. The minuscule amounts of DNA required for ODM enable typing directly from uncultured patient samples and, since each DNA fragment is analysed individually, all included species in complex bacterial mixtures can be identified and quantified. We have used ODM for bacterial plasmids where, in a single experiment, the number of different plasmids and their sizes are obtained, along with the intensity profiles that can be used for identifying the plasmids^12^. By combining the assay with Cas9 restriction we can also identify on which plasmid a specific resistance gene is located^13^. This has, for example, allowed us to trace plasmids during resistance outbreaks^14,15^ and identify clonal spread of bacteria^16^. Furthermore, the analysis can be parallelized in chips with at least ten devices, to allow simultaneous analysis of multiple samples^17^.

We have recently demonstrated that the same ODM assay can type bacteria on the species level^18^. However, to make informed clinical decisions, typing often needs to be done at a resolution that can distinguish between different pathotypes from the same bacterial species. We here demonstrate that competitive binding-based ODM can be used to identify *Escherichia coli* and *Klebsiella pneumoniae* bacteria in uncultured samples with a taxonomic resolution at the strain level. The strain-level identification is enabled by combining sensitive bioinformatics tools with a quality-assessed reference database, and we use the method to analyse *E. coli* and *K. pneumoniae* from clinical samples at three different strain-level resolutions. Since the strain groups are based on phylogenetic trees generated from a core genome alignment, they reflect the evolutionary relationship between the strains of each species. We demonstrate that the assay is directly compatible with simultaneous plasmid characterization and also with polymicrobial samples. We also show that the method can be applied to urine samples without any cultivation and conclude that ODM enables direct typing and resistance characterization of clinical samples.

## Methods

**Fig. 1:**
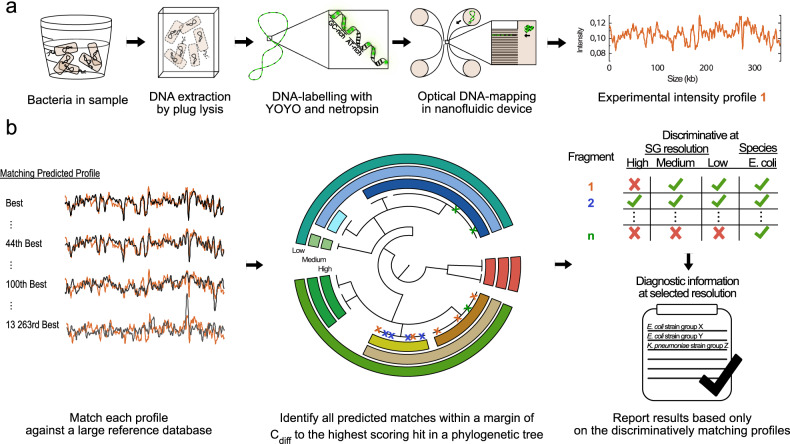
Schematic overview of high-resolution optical DNA mapping-based bacterial typing. **a** Experimental pipeline. The DNA, extracted via plug-lysis, is labelled with YOYO-1 and netropsin in a single step. The DNA is confined to nanofluidic channels and imaged using a fluorescence microscope, resulting in one experimental intensity profile per analysed DNA fragment. **b** Data analysis pipeline. Each experimental intensity profile is matched to the reference database of predicted intensity profiles. Next, the database matches are filtered for each experimental profile so that only high-scoring matches remain. Profiles are deemed to match discriminatively if all remaining matches are against a single strain group (SG) at the investigated resolution. Only the results of the discriminative profiles are reported back to the user. The result for the sample is shown as the number of discriminative fragments matching each detected SG and the proportions of all discriminative profiles matching each detected SG.

Figure 1a provides an overview of the experimental procedure and it is summarized in the following sections.

### Bacterial samples

The bacterial samples analysed were selected by clinical relevance or from their phylogenetic relationship for method evaluation (Supplementary Data 1). Bacterial strains were grown in Luria-Bertani (LB) broth, with 1.5% agar for solid medium, and stored at −80 °C in 10% DMSO stocks. The non-cultivated urine samples were collected at Karolinska University Hospital in Stockholm and used directly for DNA extraction. For samples with mixes of bacteria, each strain was cultivated separately and mixed in equal amounts before DNA extraction. In total, 25 cultured *E. coli* samples (EC1–25), nine cultured *K. pneumoniae* samples (KP1–9), six uncultured clinical urine samples containing *E. coli* (U1–6), two mixes of four *E. coli* strains each (M1-2), and three *E. coli* samples with plasmid-borne *bla*_CTX-M_ genes (P1-3) were selected for the study. The study did receive ethical approval (recordal number of the approval 2018/7273531/2) as per the Swedish Ethical Review Authority. The approval clarified that it was acceptable not to collect informed consent. We included only samples that were ready to be discarded and no additional sampling from patients was needed.

### DNA isolation

DNA extraction was carried out by enclosing bacteria in gel plugs and isolating long DNA molecules^18^. In short, 250 μL of overnight culture or 1−3 mL of a non-cultivated urine sample was pelleted and enclosed in an agarose plug (100 μL). The bacteria were lysed, treated with RNase A and proteinase K and finally washed. The lysis protocol was carried out inside the agarose plug to protect the DNA from breaking. The agarose plugs (100 μL) were melted in 20 μL of 10× CutSmart Buffer (New England Biolabs) and 78 μL of MQ- water at 70 °C for 10 min, followed by incubation at 42 °C for 10 min before the addition of 2 μL of agarase (ThermoFisher Scientific, 0.5 U/L) and a second incubation at 42 °C for at least 1 h. The DNA concentration was determined using a Qubit Fluorometer 2.0 (ThermoFisher Scientific).

### Sample preparation

The sequence-based intensity profiles for the ODM experiments were created by the addition of YOYO-1 (with an excitation of 491 nm and emission of 509 nm, Invitrogen) and netropsin (Sigma-Aldrich). 10 μL of an 0.5× TBE (Tris-Borate-EDTA, Novex, Invitrogen) solution was prepared with 1 μM (base pairs) extracted bacterial DNA, 1 μM (base pairs) λ-DNA (included as an internal size reference, 48,502 bp, Roche Biochem Reagents), 0.2 μM YOYO-1 (ratio of DNA base pairs/YOYO-1 is 10:1), and 60–120 μM netropsin (varying the ratio of netropsin:YOYO-1 between 600:1 and 300:1 optimized the emission intensity in each experiment but did not have any effect on the intensity profile matching discriminatively to the reference database), followed by incubation at 50 °C for 30 min. The DNA solution was diluted 10-fold with 88 μL of MQ-water and 2 μL of β-mercaptoethanol (used to prevent photodamaging, Sigma-Aldrich), obtaining a final buffer concentration of 0.05× TBE before loading 10 μL of the sample into a loading well of a nanofluidic chip.

The presence of antimicrobial resistance genes on plasmids in samples P1-P3 was detected using a CRISPR-Cas9 approach before DNA labelling^13^. TracrRNA and two different crRNA with sequences targeting the two main groups of the β-lactamase gene *bla*_CTX-M_, groups 1 and 9 (CCGCCGCGATGTGCTGGCTTCAG and AGAGAGCCGCCGCGATGTGCTGG, respectively) were purchased from GE Healthcare and re-suspended in 10 mM RNase free Tris- HCl buffer (Sigma-Aldrich). Guide RNA (gRNA) was created by incubating 0.33 nmol tracrRNA with 0.16 nmol of each crRNA in 1× NEBuffer 3 (New England Biolabs) and 1× (0.1 μg/μL) BSA (New England Biolabs) for 30 min at 4 °C. Next, 10 μM (0.15 nmol) of gRNA was incubated with 300 ng of Cas9 (Sigma-Aldrich), 1× NEBuffer 3 and 1× (0.1 μg/μL) BSA, at 37 °C for 15 min. Finally, 60 ng of extracted DNA from the bacterial samples was added to the mixture, followed by incubation at 37 °C for 1 h. Following the Cas9 restriction, the DNA was labelled for ODM experiments according to the slightly modified protocol 4 μM DNA (base pairs, directly from the Cas9 mixture, without purification), 1.2 μM (base pairs) λ-DNA, 2.6 μM YOYO-1 (ratio of DNA base pairs:YOYO-1 2:1), and 260 μM netropsin (netropsin:YOYO-1 ratio 100:1) followed by incubation at 50 °C for 30 min. The DNA solution was diluted 10-fold before loading 10 uL of the sample into a loading well of a nanofluidic chip.

### Nanofluidic experiments

The nanofluidic experiments were done using a nanofluidic chip with 200 parallel nanochannels spanning 500 µm in length, each with a dimension of 100 × 150 nm^2^ (height x width). The nanochannels are connected to a microchannel on each side, connecting two loading wells each. The chips were fabricated in silicon dioxide using standard micro-nanofabrication methods^19^. When recording the intensity profile, each single DNA fragment is introduced via one of the microchannels to a nanochannel using pressure-driven N_2_ gas flow, forcing the molecule to stretch out. When the DNA fragment is stretched out in the nanochannel, the DNA was imaged using a Zeiss AxioObserver.Z1 fluorescence microscope with a 1.6x optovar, 63x oil immersion objective (NA = 1.46 Zeiss) and an Andor iXon EMCCD camera. For each DNA fragment at least 20 timeframes with an exposure time of 200 ms were recorded. If the stretch factor, calculated from the reference molecule λ-DNA, was less than 0.2 nm/bp the data was discarded.

### Data analysis

An overview of the principle of the data analysis can be seen in Fig. 1b. For each recorded DNA fragment, an experimental intensity profile was generated from at least 20 timeframes via time-averaged kymographs. The generation of time-averaged kymographs is explained in detail in the Supporting Data of a previous study^20^. Next, all experimental intensity profiles were compared to a reference database of in silico predicted profiles that was constructed in the following way. To obtain high-quality genome assemblies, all complete bacterial genomes were downloaded from NCBI’s RefSeq database (2019-07-17). Since our reference database was developed for species and strain identification, we aimed to include only complete chromosomal sequences and, therefore, removed sequences shorter than 500 kb or with the words “plasmid” in the FASTA header. Similarly, sequences with dubious taxonomic classification – i.e. with “Candidatus” or “bacterium” in the FASTA header – were also removed. This left 13,024, most likely complete and chromosomal, sequences with a FASTA header containing a full bacterial species name (2697 different species). For a full list of included accession numbers, see Supplementary Data 2. All sequences were converted into predicted intensity profiles by calculating the binding probabilities of YOYO-1 along the DNA molecule in the presence of Netropsin, and adding camera effects to mimic the effects of imaging in a nanochannel using a fluorescence microscope^20–22^.

Of the reference genome sequences, 867 belonged to the *Escherichia coli*/*Shigella* group and 348 to *Klebsiella pneumoniae*. Due to their genetic similarity on the chromosomal level, *E. coli* and *Shigella* spp. were treated as a single species in this study^23^. These references were assigned to strain groups (SGs), i.e. taxonomic groups at a higher resolution than species, in the following way. Firstly, the sequences were annotated using Prokka (version 1.12, default parameters) and the resulting gff-files were used as input to Roary (version 3.13.0, parameters –e –p 20) to identify the core genomes for each of the species^24,25^. Since the *E. coli/Shigella* group is a diverse group of bacteria, we used additional parameters (–g 100000 –cd 95) to avoid a very small core genome. In *E. coli/Shigella*, 1697 genes were classified as core genes (present in 95% of the analysed genomes) and in *K. pneumoniae*, there were 2 804 core genes (present in 99% of the analysed genomes). Phylogenetic trees were constructed using the core genome alignments from Roary with FastTree (version 2.1.11, double precision) and the trees were visualized using iTol with midpoint rooting^26,27^. An R-script was used to read the Newick tree files (package phytools version 0.6–99) and divide the trees into SGs based on branch lengths (package dendextend version 1.13.4; parameter *h* = 0.03/0.015/0.008/0.003 for *E. coli* SG_Low_/SG_Medium_/SG_High_/ SG_Ultra-High_ and *h* = 0.008/0.007/0.003 for *K. pneumoniae*)^28,29^. See Supplementary Figure 1 and Supplementary Fig. 2 for illustrations of the trees and SG schemes. For a given threshold branch length (parameter h), each reference genome of *E. coli/Shigella* and *K. pneumoniae* was assigned to a single SG. A short branch length created many SGs and, consequently, an SG scheme of a high taxonomic resolution and vice versa. For the selected resolutions, the 867 *E. coli* reference genomes in the database were divided into 88 (SG_High_), 34 (SG_Medium_), and 10 (SG_Low_) SGs, respectively. At SG_High_, all reference genomes of the globally disseminated *E. coli* sequence type (ST) 131 formed their own SG. Analogously for *K. pneumoniae*, the 348 reference genomes were divided into 62 (SG_High_), 22 (SG_Medium_), and 11 (SG_Low_) groups, respectively. Here, the clinically important clonal complexes CC14, CC147, CC258, and CC307 all formed distinct SGs at SG_High_, except for the SG containing CC258, which also contained a single additional genome (Supplementary Data 3 and Supplementary Data 4). In addition, all *E. coli/Shigella* and *K. pneumoniae* sequences were typed using MLST^30–32^ and the *E. coli* phylogroups were determined using EZClermont^33^. For a full list of all *E. coli/Shigella* and *K. pneumoniae* reference genomes, their SG assignment for the three investigated SG resolutions, their sequence type (ST), and, for *E. coli*, their phylogroup, see Supplementary Data 3.

The comparison between the experimental intensity profiles and the reference database of predicted profiles^20^ results in a similarity score – based on the Pearson correlation coefficient – for the optimal alignment of each experimental profile against each reference. The reference is stretched to the experimentally measured nanometer per base pair stretch factor determined using the reference molecule λ-DNA, the alignment allows for fluctuations of the DNA molecule (with re-scaling factors 90–110% in steps of 2.5%), and the experimental profile is slid along the reference (in both directions) to identify the optimal alignment of the profile pair. The scores are used to identify experimental profiles that are informative on the taxonomic level of interest, i.e. they match discriminatively to a single taxonomic group. We have previously shown that this method is effective in typing bacterial samples to the species level^18^, and we here apply the same principle to varying SG resolutions. Firstly, all intensity profiles that are too short are removed (rescaled length <L_min_). For each remaining experimental profile, the best matches – defined as the highest-scoring match and any match within C_diff_ of the highest score – are kept. If the highest score for the profile is above a threshold, C_thresh_, and if all the best matches are to a single SG, then the profile is deemed to match discriminatively to that SG. Only the results of the discriminative profiles are reported back to the user. There are thus four parameters that affect the results: the minimum allowed profile length (L_min_, rescaled length), the width of the score range of the best matches for a profile (C_diff_), the minimum allowed maximum score of a profile (C_thresh_), and the taxonomic resolution of interest. The parameter L_min_ was set to 250 pixels and C_thresh_ was set to 0.5 throughout this study based on the results of our previous work^18^. The effect of the remaining two parameters was tested in terms of the proportion of all profiles that matched any SG discriminatively and the sensitivity measured as the true positive rate (TPR) – i.e., the proportion of the discriminative profiles that matched the correct SG. The correct SG was determined as follows. All samples were included in a phylogenetic tree together with all reference genomes of the same species using the same methods and parameters as described above. For each sample, the SG assignment of the closest reference genome in the tree was identified as the correct one.

Analysis of potential plasmid fragments was performed using an automated, custom-written Matlab-based program (see Data Availability)^13,18^. In short, the extensions of the fragments were extracted from the time-averaged kymographs and the fragments were first grouped based on their length. For a given size group, the associated intensity profiles were aligned and clustered based on similarity using a correlation coefficient for their alignment to one another (exemplified in Supplementary Fig. 3). The presence of the target gene was verified by analysing whether most double-strand breaks occurred at the same position in a similarity cluster (typical correlation coefficient threshold of 0.7). This indicates that the cuts are caused by Cas9 cuts at the target gene location, as opposed to being caused by mechanical forces or photonicking (exemplified in Supplementary Fig. 3).

### Whole-genome sequencing of selected samples

Whole-genome sequences of the samples were used to assign each sample to its correct SG for each of the tested SG resolutions to confirm the ODM data. The *E. coli* samples EC3–4, EC7-11, and EC18 had been previously sequenced by Fröding et al.^34^ (assembly accession numbers GCA_013171465.1, GCA_013172225.1, GCA_013172765.1, GCA_013174205.1, GCA_013176495.1, GCA_013176595.1, GCA_013176845.1, and GCA_013176885.1) and the *K. pneumoniae* samples KP2–3, KP7, and KP9 by Koskinen et al.^35^ (GCA_016056235.1, GCA_015831445.1, GCA_016056215.1, and GCA_015831545.1). These assemblies were all downloaded from GenBank. The samples KP1, KP4-6, and KP8 were sequenced and assembled using the same method as the other *K. pneumoniae* samples^35^. The remaining samples were sequenced for this study in the following way. For plasmid sample P1, the DNA extraction was done using the Qiagen EZ1 DNA Tissue Kit and EZ1 Advanced XL instrument followed by paired-end sequencing with a read length of 300 bp on an Illumina NovaSeq using the Nextera library prep kit. For samples Mix2.1, Mix2.2, EC20, EC22, and EC24 the DNA was extracted using the robot PSS magLEAD 12gC with the Magtration reagent MagDEA Dx SV reagent kit. Sequencing was performed on an Ion Torrent PGM System for samples Mix2.1, Mix2.2, and EC22 (400 bp, no size selection) and an Ion Torrent Ion S5 XL system for samples EC20 and EC24 (300 bp, autosize selection) using the Ion Xpress Plus Fragment Library Kit for AB Library Builder System. The rest of the samples – *E. coli* samples EC1–2, EC5–6, EC12–17, EC19, EC21, EC23, EC25, Mix1.1, all urine samples (U1–6), and plasmid samples P2–3 – were extracted for total DNA using the Illumina Epicenter MasterPure^TM^ DNA purification kit according to the manufacturer’s instructions and sequencing was performed on an Illumina MiSeq using the Nextera XT library prep kit and sequenced with a paired-end read length of 300 bp. The *E. coli* samples EC1–2, EC5–6, EC12, EC17, EC19–25, and all urine samples (U1–U6) were analyzed with CLC Genomics Workbench (Qiagen, v21) by de novo assembly. Multilocus sequence typing was performed from de novo contigs using the CLC Microbial Genomics module. The sequencing data for *E. coli* samples EC13–EC16 and all plasmid samples (P1–3) were first trimmed for adaptors and low-quality ends using Trim Galore! (https://www.bioinformatics.babraham.ac.uk/projects/trim_galore/; v. 0.4.3, parameters –stringency 3 –paired –retain_unpaired) and then assembled using SPAdes (v. 3.14.1, parameters –isolate –cov-cutoff 5).

### Reporting summary

Further information on research design is available in the Nature Portfolio Reporting Summary linked to this article.

## Results & discussion

### Overview of the proposed method

Here we show that competitive binding-based ODM can be used for accurate typing of *E. coli* and *K. pneumoniae* at the strain level for uncultured samples (Fig. 1). Long DNA fragments are isolated directly from patient samples in a simple agarose-based lysis and purification step, fluorescently stained in a single step using YOYO-1 and netropsin, and finally, intensity profiles are recorded (Fig. 1a). The intensity profiles are then matched to a reference database of in silico predicted intensity profiles and similarity scores are returned (Fig. 1b). The database used here is generated from high-quality, complete bacterial chromosome sequences (13,024 sequences from 2697 species, Supplementary Data 2). Furthermore, the reference database is divided into strain groups (SGs), i.e. taxonomic groups at a higher resolution than species. Our SGs schemes are defined based on phylogenetic trees generated from a core genome alignment and, therefore, reflect the evolutionary relationship between the strains or subtypes of each species. The SGs are formed by cutting each tree at a given branch length, which defines the size and diversity of the SGs and hence the taxonomic resolution (Fig. 1b). Only the highest-scoring matches between an experimental profile and the reference profiles in the database are retained. An experimental profile is deemed discriminative at a given resolution if all the high-scoring reference matches belong to the same SG. Only discriminative profiles are used for the strain-level identification, and the remaining profiles are discarded since they are deemed uninformative at the taxonomic resolution of interest. We here focus on three strain-level resolutions: SG_High_, SG_Medium_, and SG_Low_ (Supplementary Figure 1 and Supplementary Fig. 2, see Methods for details). At present, the time from patient sample to the final result is around 7 h: 4 h DNA extraction, 40 min DNA processing and labelling, 1 h nanofluidic imaging, and 1 h data analysis, which can be further optimized. This paper provides a proof-of-concept description of the method and the full analysis has, therefore, not yet been optimized in terms of time and cost. The nanofluidic devices used are custom-built in the clean-room facilities at the Chalmers University of Technology, so the protocol is currently not applicable in labs that cannot fabricate such devices. All other parts of the protocol are general and only commercially available reagents are used. Furthermore, all data analysis protocols are freely available online and the experiments are straightforward to perform.

### Parameter optimization and method performance using cultured samples

**Fig. 2:**
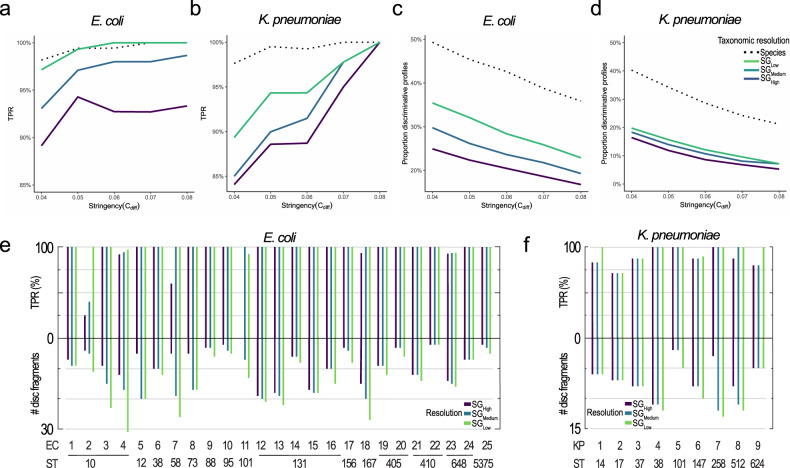
Parameter evaluation and typing results for pure cultured *E. coli* and *K. pneumoniae* isolates. **a**–**d** The effects of different filtering stringencies (parameter C_diff_) on the average true positive rate (TPR) and the average proportion of discriminative profiles were assessed using 25 E. coli samples (**a**, **c**) and nine K. pneumoniae samples (**b**, **d**). The method was evaluated on the three investigated strain-level taxonomic resolutions – SG_Low_, SG_Medium_ and SG_High_ – as well as on the species level. **e** The number of discriminative intensity profiles and true positive rate (TPR) for each cultivated pure E. coli sample for the three investigated taxonomic resolutions, SG_High_, SG_Medium_ and SG_Low_. **f** The number of discriminative intensity profiles and true positive rate (TPR) for each cultivated pure K. pneumoniae sample for the three investigated taxonomic resolutions, SG_High_, SG_Medium_ and SG_Low_.

We first validated the ODM method on cultured *E. coli* (n = 25) and *K. pneumoniae* (*n* = 9) isolates. The method performance is assessed by measuring the sensitivity – estimated by the average true positive rate (TPR, Fig. 2a, b) – and the average proportion of discriminative profiles (Fig. 2c, d) for different stringency thresholds in the database matching (defined by parameter C_diff_, see Methods for details). By increasing the stringency, the sensitivity generally increases for all resolutions, while the average proportion of discriminative profiles decreases. There is, consequently, a trade-off between obtaining high sensitivity and including as many profiles as possible – which ultimately comes down to the total number of DNA fragments analysed. In situations where data is abundant, the stringency of the matches to the reference database can be increased to ensure an even higher TPR. On the other hand, if time is of the essence and/or data is scarce, the stringency can be lowered, or a lower taxonomic resolution can be used and, thus, the method is adaptable to different clinical settings and applications. The average sensitivity is high for all tested SG resolutions and levels of stringency (*E. coli* TPR ≥ 89%, *K. pneumoniae* TPR ≥ 84%; Fig. 2a, b). As expected, the sensitivity was highest at SG_Low_ which, for *E. coli*, was on par with the sensitivity at the species level (TPR ≥ 97%). Both the sensitivity and the proportion of discriminative profiles were somewhat lower for *K. pneumoniae* (TPR ≥ 89%), likely due to less genetic diversity within the species compared to *E. coli*^36^, which makes it harder to distinguish between different groups. All subsequent results are based on the stringency set to C_diff_ = 0.05, which results in high sensitivity while still retaining enough data. This stringency results in an average TPR of 99.3/97.1/94.3% for *E. coli* and 94.3/90.0/88.6% for *K. pneumoniae* at SG_Low_/SG_Medium_/SG_High_, respectively, which can be compared to 99.4% (*E. coli*) and 99.5% (*K. pneumoniae*) at the species level. Some variation between individual samples is observed, both in sensitivity and the number of discriminative profiles (Fig. 2e, f and Supplementary Data 1). Certain samples are comparatively more challenging to type, possibly due to fewer genomes from that SG in the reference database or false positives caused by matches against chromosomal mobile genetic elements. Nevertheless, the TPR is ≥ 90% for 22 of the 25 *E. coli* samples and ≥ 80% for 8 of the 9 *K. pneumoniae* samples for all three SG resolutions, indicating an overall high sensitivity.

Analogously to many other typing methods, the diversity and accuracy of the reference database will have an impact on the results. Ideally, the database would include representative genomes of all relevant bacteria – including commensals that may be present in clinical samples. In this study, we based our reference database on NCBI RefSeq which is biased towards pathogenic bacteria, which, in theory, could lead to false positives of closely related non-pathogenic isolates. However, the overall high performances discussed above show that our database is sufficiently comprehensive for strain typing of clinically relevant *E. coli* and *K. pneumoniae* strains. With an increasing number of sequenced genomes, the accuracy of the method will likely continue to improve. Furthermore, updated versions or tailored reference databases can be built using any set of reference sequences and our tool for in silico profile prediction (see Code Availability)^20,21^. The SGs could be defined either manually with expert knowledge or using our approach of pruning phylogenetic trees to form groups of genomes that share a similar core genome. The database and SG schemes can, hence, be adjusted to other, clinical or non-clinical, applications.

### Polymicrobial samples and urine samples

**Fig. 3:**
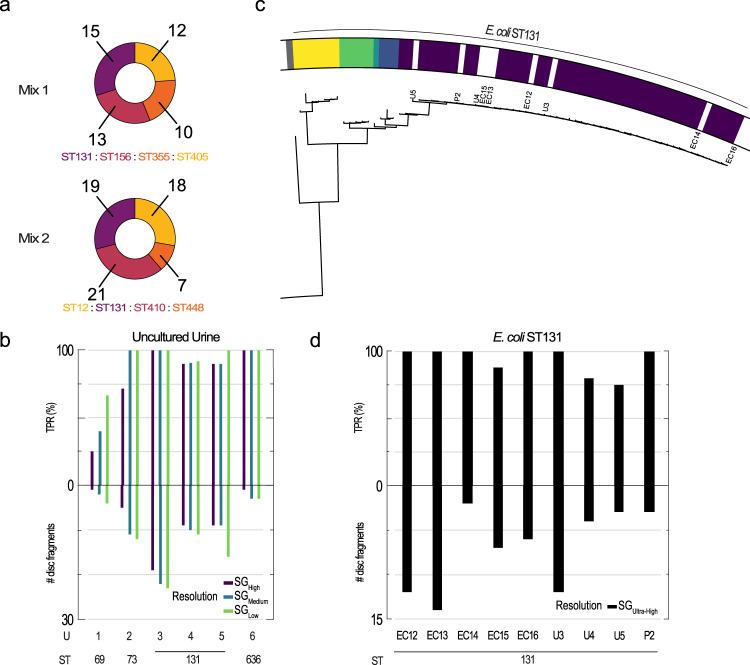
Typing results for mixes, urine samples, and ST131 samples with ultra-high resolution. **a** The distribution of discriminative strain group (SG) matches at SG_Medium_-resolution for the two mixed samples, each containing four different *E. coli* strains in equal proportions. The number of profiles matching discriminatively to each identified SG is indicated next to the chart. **b** The number of discriminative intensity profiles and true positive rate (TPR) for six non-cultivated urine samples at the three investigated strain-level taxonomic resolutions, SG_High_, SG_Medium_ and SG_Low_. **c** The ST131 branch of the phylogenetic tree (full tree in Supplementary Fig. 1) includes the nine ST131 samples (white) and the closest non-ST131 genome (far left in grey). The five SGs for this ultra-high resolution are marked (yellow-purple). **d** The number of discriminative intensity profiles and true positive rate (TPR) for the nine ST131 samples plotted at ultra-high resolution (SG_Ultra-High_), corresponding to the five SGs of the ST131 branch.

A clear benefit of the ODM-based typing method is that each intensity profile is individually assigned to a taxonomic group and, hence, even bacteria in mixed polymicrobial samples are easily identified. In two mixtures, each with four different *E. coli* strains in equal proportions, we obtain an average TPR of 100/100/91% at SG_Low_/SG_Medium_/SG_High_ (Fig. 3a), showing that strain-level typing can be done with high sensitivity for bacterial mixtures of the same species. The number of different bacteria that can be identified in a single sample depends solely on the number of fragments that are collected, and their relative abundance. The detection limit is all based on the total number of DNA fragments analyzed and can thus be adapted to the sample at hand. Furthermore, the assay requires minuscule amounts of DNA (loaded DNA: 10 picomoles, concentration in base pairs ≥ 500 nM; analysed DNA: 10 attomoles in base pairs)^18^. This enables the analysis of samples without prior cultivation, which is a time-consuming step in bacterial typing. To illustrate this, bacteria from six urine samples from patients with *E. coli* infections are enclosed in gel plugs for DNA extraction and analysis. The results are in agreement with the cultured isolates with an average TPR of 93/87/79% at SG_Low_/SG_Medium_/SG_High_ (Fig. 3b). Indeed, all individual urine samples have a TPR of ≥ 90% at the three tested SG resolutions, except U1 (ST69, Fig. 3b) which still had a 100% TPR at the species level.

### Ultra-high resolution typing of *E. coli* ST131

To further explore the resolution of the method, we investigate if different clonal lineages of *E. coli* ST131 can be distinguished. ST131 is a globally disseminated pathogenic strain that has spread quickly, is associated with extensive antimicrobial resistance and severe infections, and where different clades exhibit specific virulence and resistance phenotypes^37,38^. We use an ultra-high SG resolution for *E. coli* that divides the 61 ST131 reference genomes in the database into five SGs (Fig. 3c) and, for reference, the 867 *E. coli* reference genomes into 180 SGs. The resulting sensitivity is high with an average TPR of 94% and six of the nine samples having a TPR of 100% (Fig. 3d). This shows that our method can accurately type bacteria, even at a taxonomic resolution that is higher than the traditional MLST level.

### Simultaneous plasmid characterization and bacterial typing

**Fig. 4:**
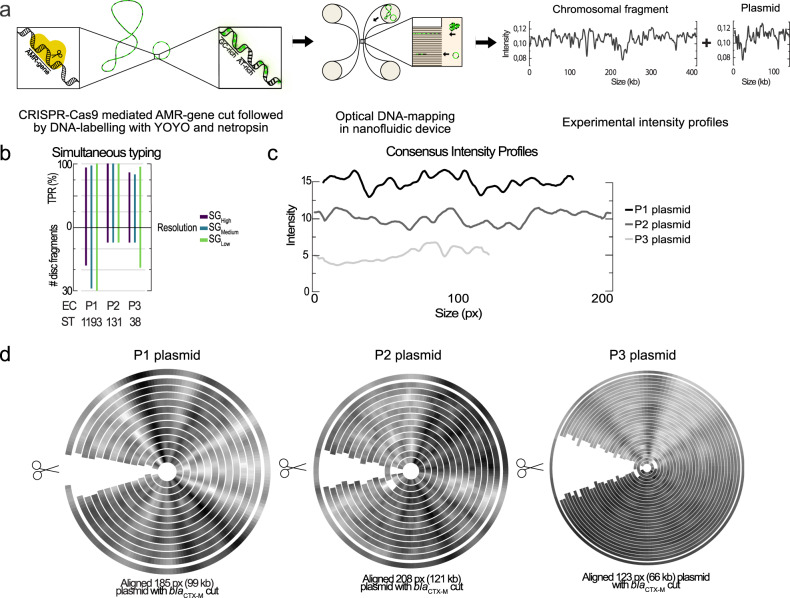
Simultaneous bacterial typing and plasmid characterization. **a** Experimental pipeline. DNA is extracted via plug-lysis and the gene of interest is cut by Cas9 followed by YOYO-1 and netropsin labelling. The labelled DNA sample, containing both chromosomal and plasmid DNA, is confined to nanofluidic channels and imaged using a fluorescence microscope, resulting in one experimental intensity profile per DNA fragment. **b** Bacterial typing using profiles longer than 250 pixels (~135 kb). The bar charts show the true positive rate (TPR) and the number of discriminative intensity profiles for samples P1, P2 and P3 at the three strain group (SG) resolutions, SG_High_, SG_Medium_ and SG_Low_. **c** The intensity profiles for the consensuses from the plasmids from P1, P2, and P3 shifted vertically for clarity. **d** Detection of plasmid-borne *bla*_*CTX*-M_ group 1 and 9 genes. In the circular plot, each ribbon represents individual DNA fragments and their brightness corresponds to the intensity profile. The profiles have been aligned and the consensus of all the profiles is included as the outermost ribbon. Linearization at the same position indicates a cut by Cas9 and verifies the presence of the target gene (see examples of randomly linearized in Supplementary Fig. 3). The three circular plots show linearization by Cas9 for the 99 kb plasmid in sample P1 (12/12 profiles linearized at the same position), 121 kb plasmid in sample P2 (12/13) and for the 66 kb plasmid in sample P3 (26/28).

Finally, we extended the protocol to combine high-resolution bacterial typing with plasmid characterization and resistance gene detection in a single experiment. By using Cas9, the presence of antibiotic-resistance genes on plasmids can be detected with high accuracy (Fig. 4a)^12,14,15^. The assay is based on finding Cas9 cuts at the same position in plasmids with identical intensity profiles, which indicates the presence of a specific gene (see Supplementary Fig. 3 for a detailed example). As proof of principle, we combine the typing assay for bacteria with guide RNA targeting Cas9 to β-lactamase genes belonging to CTX-M groups 1 and 9^39^. In samples P1, P2, and P3 we identify the correct bacterial SG with an average TPR of 98/94/93% at SG_Low_/SG_Medium_/SG_High_, respectively (Fig. 4b). Simultaneously, we identified one plasmid in each sample (99 kb, 121 kb, and 66 kb in size, respectively) carrying the *bla*_CTX-M_ gene. The three *bla*_CTX-M_ plasmids found are distinctly different both in length and sequence (Fig. 4c). The presence of the *bla*_CTX-M_ gene on the plasmids is verified by Cas9-induced linearization for the 99 kb plasmid in sample P1 (12/12 profiles linearized at the same position), 121 kb plasmid in sample P2 (12/13) and for the 66 kb plasmid in sample P3 (26/28) (Fig. 4d). The presence of these *bla*_CTX-M_ genes is confirmed by sequencing. This is the first example, to the best of our knowledge, of a method that is independent of culturing and that can directly identify a specific bacterial isolate at the strain level and link it with a plasmid carrying resistance genes. This will open up for analysis of both bacterial and plasmid transmission between and within patients and can also potentially be used to identify endemic clones in the microbiome.

## Conclusion

In conclusion, we here introduce an ODM-based method for typing bacteria at the strain level and demonstrate its high performance for *E. coli* and *K. pneumoniae*. The taxonomic resolution of the method can be tuned for the application at hand by changing the variability within the SGs and, consequently, their size. Since our SG scheme is based on core genome alignments, it reflects the underlying evolutionary relationship within the species, making it possible to identify bacteria that are from clinically challenging lineages. We successfully identify isolates up to a taxonomic resolution corresponding to dividing the globally disseminated high-risk lineage *E. coli* ST131 into five SGs which would clearly define clades of concern within this important *E. coli* strain group. The method has several important features making it attractive as a future tool for diagnosing bacterial infections. The protocol used for the extraction of long DNA fragments is based on methods already widely used in microbiological laboratories and we have previously shown that it applies to both Gram-positive and Gram-negative bacteria for species-level identification^18^. The labelling method relies completely on non-covalent interactions with DNA, does not depend on primers or probes, and, therefore, works for any bacterial species and is impervious to allelic variation of the particular infecting isolate. The single-molecule aspect of the method means that complex mixtures of bacteria can be readily analysed. Furthermore, any potential contamination by patient DNA will not interfere with the analysis as any human DNA is highly unlikely to match discriminatively to any bacterial species. Due to the low amount of DNA needed for the analysis, the method can avoid the time-consuming step of cultivation. We also show that the method can be combined with plasmid identification and identification of resistance genes in the same experiment. This is not trivial for existing techniques and is, for example, crucial for finding epidemic clones in the microbiome, which is central to infection control. To summarize, we present a versatile method that enables new possibilities for diagnosing bacterial infections and identifying particularly worrisome pathogens.

## Supplementary information

Supplementary Information

Supplementary Data 1

Supplementary Data 2

Supplementary Data 3

Supplementary Data 4

Supplementary Figure 1

Supplementary Figure 2

Description of Additional Supplementary Files

Reporting Summary

## Data Availability

Raw kymographs of individual molecules from the optical DNA mapping have been deposited with figshare and can be accessed at: 10.6084/m9.figshare.c.5760860.v1^40^. A list of NCBI accession numbers of all sequences used to build the reference database is available as Supplementary Data 2. Supplementary Data 3 lists all *Escherichia coli/Shigella* spp. and *Klebsiella pneumoniae* genomes included in the reference database and their assigned strain group (SG) for the tested strain-level taxonomic resolutions: SG_Low_, SG_Medium_, SG_High_, and, only for *E. coli*, *SG*_*Ultra-High*_. For comparison to existing strain-level groupings, the lists also include type (ST) and, only for *E. coli*, their Clermont’s phylogroup, for each reference sequence. A list of all the included STs for each SG at each of the tested strain-level resolutions is available as Supplementary Data 4. A list of all the analysed samples together with their species, sequence type, sequencing data accession number, references to relevant figures in the main text, and the numbers of experimental intensity profiles are available as Supplementary Data 1. Raw sequencing data generated for this project have been submitted to NCBI SRA and all studied samples are linked to BioProject accession number PRJNA774113^41^. All other data is available upon reasonable request from the corresponding author. Figure 2a–f, Fig. 3a, b, d, and Fig. 4b was generated using the data in Supplementary Data 1. Figure 3c was generated from the reference genomes listed in Supplementary Data 2 and the sequenced samples linked to the BioProject accession number PRJNA774113^41^. Figure 4c, d was generated using the kymographs for plasmids, where the kymographs and experimental settings (KymographInfo.xlsx) can be accessed at 10.6084/m9.figshare.c.5760860.v1^40^.

## Code availability

The code for consensus intensity profile analysis is contained in the lldev package and deposited at 10.5281/zenodo.5718208^42^. The code for generating intensity profiles and aligning them against in silico predicted intensity profiles is contained in the HCA package and deposited at 10.5281/zenodo.5718183^22^. The code for analysing the alignment results and identifying intensity profiles that match discriminatively to a single taxonomic group is deposited at 10.5281/zenodo.5898280^43^.
