# Supplementary material for: Strain-level bacterial typing directly from patient samples using optical DNA mapping
Source: Commun Med (Lond). 2023 Feb 23;3:31. doi: 10.1038/s43856-023-00259-z (PMC9950433; doi:10.1038/s43856-023-00259-z)
Supplement: Supplementary file 1 — Supplementary Information [file 43856_2023_259_MOESM1_ESM.pdf]

SUPPLEMENTARY INFORMATION

TITLE

Strain-level bacterial typing directly from patient samples using optical DNA mapping

AUTHORS

My Nyblom†[1], Anna Johnning†[2,3,4], Karolin Frykholm[1], Marie Wrande[5], Vilhelm Müller[1], Gaurav Goyal[1], Miriam Robertsson[1], Albertas Dvirnas[6], Tsegaye Sewunet[7], Sriram KK[1], Tobias Ambjörnsson[6], Christian G. Giske[7,8], Linus Sandegren[5], Erik Kristiansson[2,4,]* and Fredrik Westerlund[1,]*

AFFILIATIONS

[1] Department of Life Sciences, Chalmers University of Technology, Gothenburg, 412 96, Sweden

[2] Department of Mathematical Sciences, Chalmers University of Technology & University of Gothenburg, Gothenburg, 412 96, Sweden

[3] Department of Systems and Data Analysis, Fraunhofer-Chalmers Centre, Gothenburg, 412 88, Sweden

[4] Centre for Antibiotic Resistance Research (CARe) at University of Gothenburg, Gothenburg, 405 30, Sweden

[5] Department of Medical Biochemistry and Microbiology, Uppsala University, Uppsala, 751 23, Sweden

[6] Department of Astronomy and Theoretical Physics, Lund University, Lund, 223 62, Sweden

[7] Department of Laboratory Medicine, Karolinska Institutet, Stockholm, 141 86, Sweden

[8] Department of Clinical Microbiology, Karolinska University Hospital, Stockholm, 171 76, Sweden

* To whom correspondence should be addressed.

FW: Tel: +46 31 772 3049; Email: fredrik.westerlund@chalmers.se

EK: Tel: +46 31 772 3521; Email: erik.kristiansson@chalmers.se

† Joint First Authors

SUPPLEMENTARY FIGURES

Supplementary Figure 1. See file Supplementary_Figure_1.pdf.

Escherichia coli tree. The phylogenetic tree of the *Escherichia coli/Shigella* spp. genomes in the reference database. The three outer circles represent the SG assignment of each reference genome for each of the three investigated strain-level taxonomic resolutions (from outermost going inwards: SG_{Low}, SG_{Medium}, and SG_{High}) and the innermost circle represents the sequence type (black if the ST is represented by few genomes).

Supplementary Figure 2. See file Supplementary _Figure_2.pdf.

Klebsiella pneumoniae tree. The phylogenetic tree of the *Klebsiella pneumoniae* genomes in the reference database. The three outer circles represent the SG assignment of each reference genome for each of the three investigated strain-level taxonomic resolutions (from outermost going inwards: SG_{Low}, SG_{Medium}, and SG_{High}) and the innermost circle represents the sequence type (black if the ST is represented by few genomes).

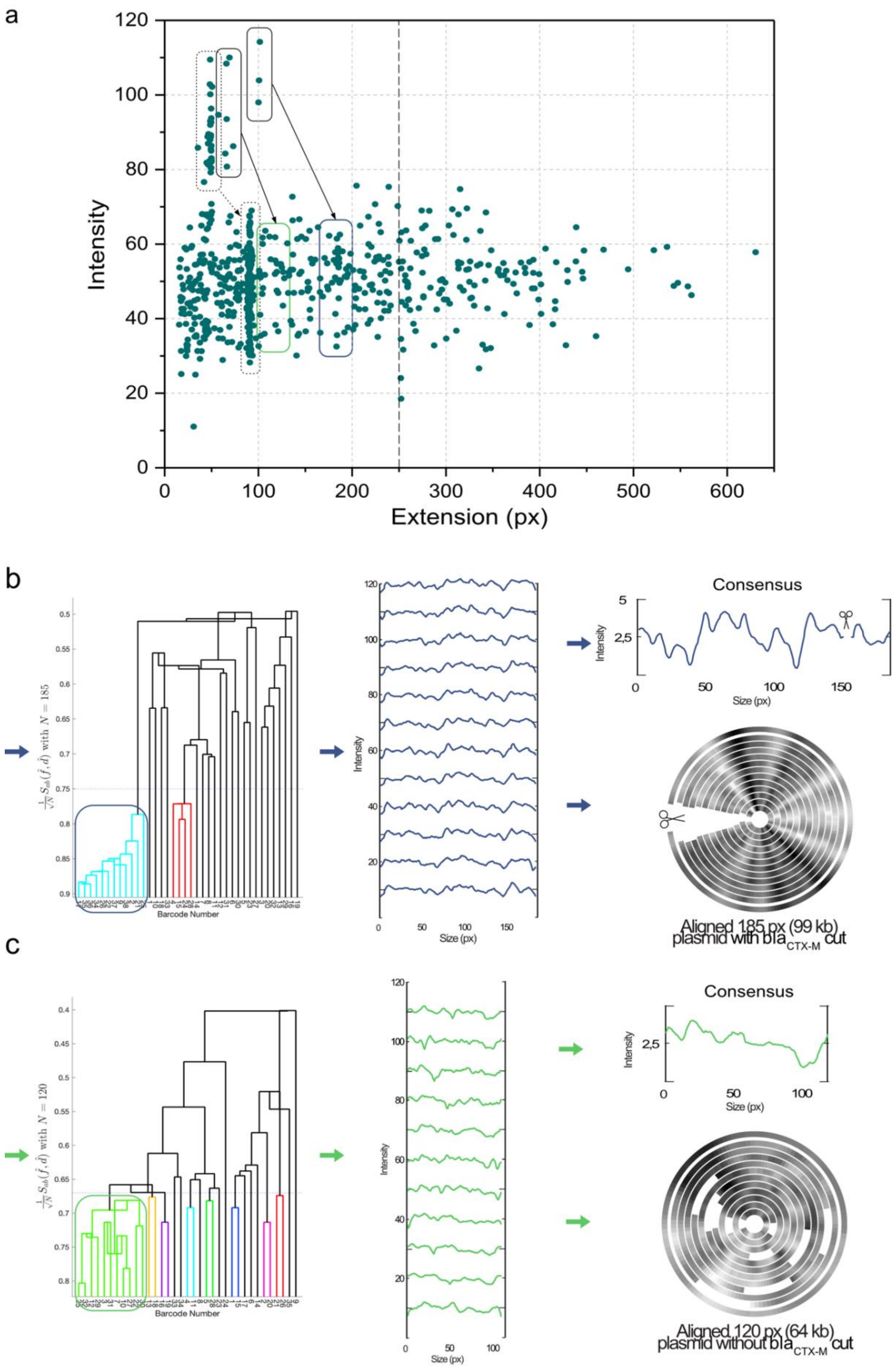

a
Intensity
Extension (px)
b
$\frac{1}{\sqrt{N}} S_{ab}(\hat{f}, \hat{d})$ with $N = 185$
Barcode Number
Intensity
Size (px)
Consensus
Aligned 185 px (99 kb) plasmid with bla$_{CTX-M}$ cut
c
$\frac{1}{\sqrt{N}} S_{ab}(\hat{f}, \hat{d})$ with $N = 120$
Consensus
Aligned 120 px (64 kb) plasmid without bla$_{CTX-M}$ cut

Supplementary Figure 3. Example of plasmid clustering for sample P1 to determine the presence of the *bla*CTX-M gene. (a) Emission intensity plotted against the extension for each experimental intensity profile. Profiles longer than 250 pixels (dotted vertical line, ~135 kb long) are used for typing the bacteria. λ-DNA (dotted rectangles) is used for size estimation. Intact circular plasmids are identified from groups with high emission intensity and similar extensions (black rectangles). The corresponding linear fragments, at approximately double the extension, (green and blue rectangles) are extracted and clustered. Intensity profiles that form clusters of high similarity are aligned to show the position of linearization. (b) All linearized fragments at 185±20 pixels (blue rectangle in (a)), from the circular plasmids at around 100 pixels, were clustered based on the similarity of the intensity profiles. Two clusters (below a similarity score threshold of 0.75) were formed in the dendrogram: twelve blue fragments (blue rectangle in (b)) and four red fragments. The red fragments were identified as dimers of λ-DNA. The intensity profiles from the blue rectangle were plotted with a shifted intensity of +10 for each fragment for clarity, and the intensity profiles are fairly similar so that a clear consensus of the plasmids can be created. All intensity profiles are then visualized individually in the circular plot, with the consensus as the outermost circle. In the circular plot, all profiles (12/12) are linearized at the same position, verifying the presence of the *bla*CTX-M gene. (c) All linearized fragments at 120±20 pixels (green rectangle in (a)), from the circular plasmids at around 65 pixels, were clustered based on the similarity of the intensity profiles. One larger cluster (below a similarity score threshold of 0.67) was formed in the dendrogram, consisting of eleven fragments (green rectangle in (c)). The intensity profiles from the green rectangle were plotted with a shifted intensity of +10 for each fragment for clarity, but the intensity profiles are not similar. All intensity profiles are then visualized individually in the circular plot, with the consensus as the outermost circle. In the circular plot, the cut sites for the linearized plasmid are randomly distributed, indicating mechanically or light-induced double-strand breaks and no presence of the *bla*CTX-M gene.
