# Supplementary figures and images for: Strain-level bacterial typing directly from patient samples using optical DNA mapping
Source: Commun Med (Lond). 2023 Feb 23;3:31. doi: 10.1038/s43856-023-00259-z (PMC9950433; doi:10.1038/s43856-023-00259-z)

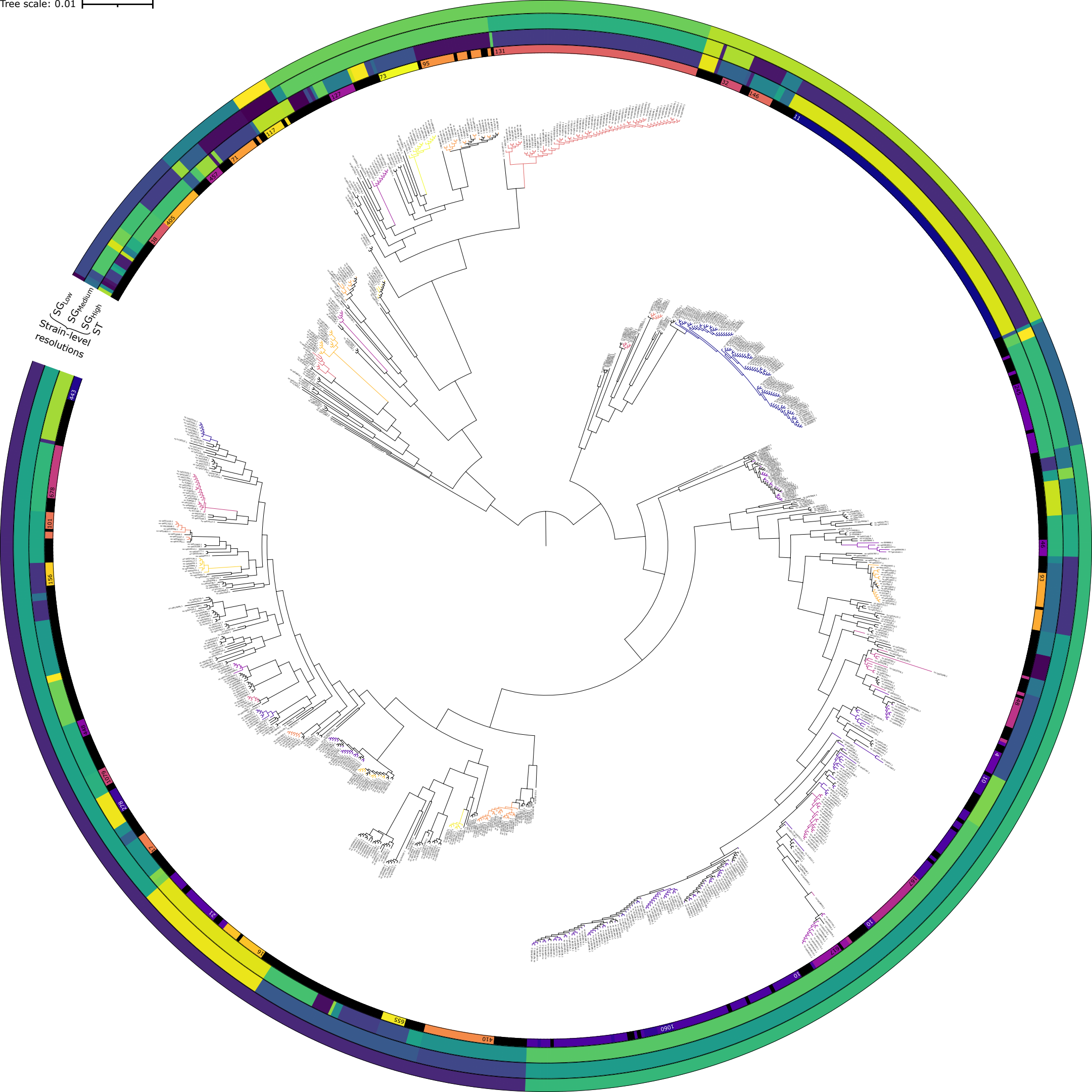

Supplement: Supplementary file 6 — Supplementary Figure 1 [file 43856_2023_259_MOESM6_ESM.pdf]

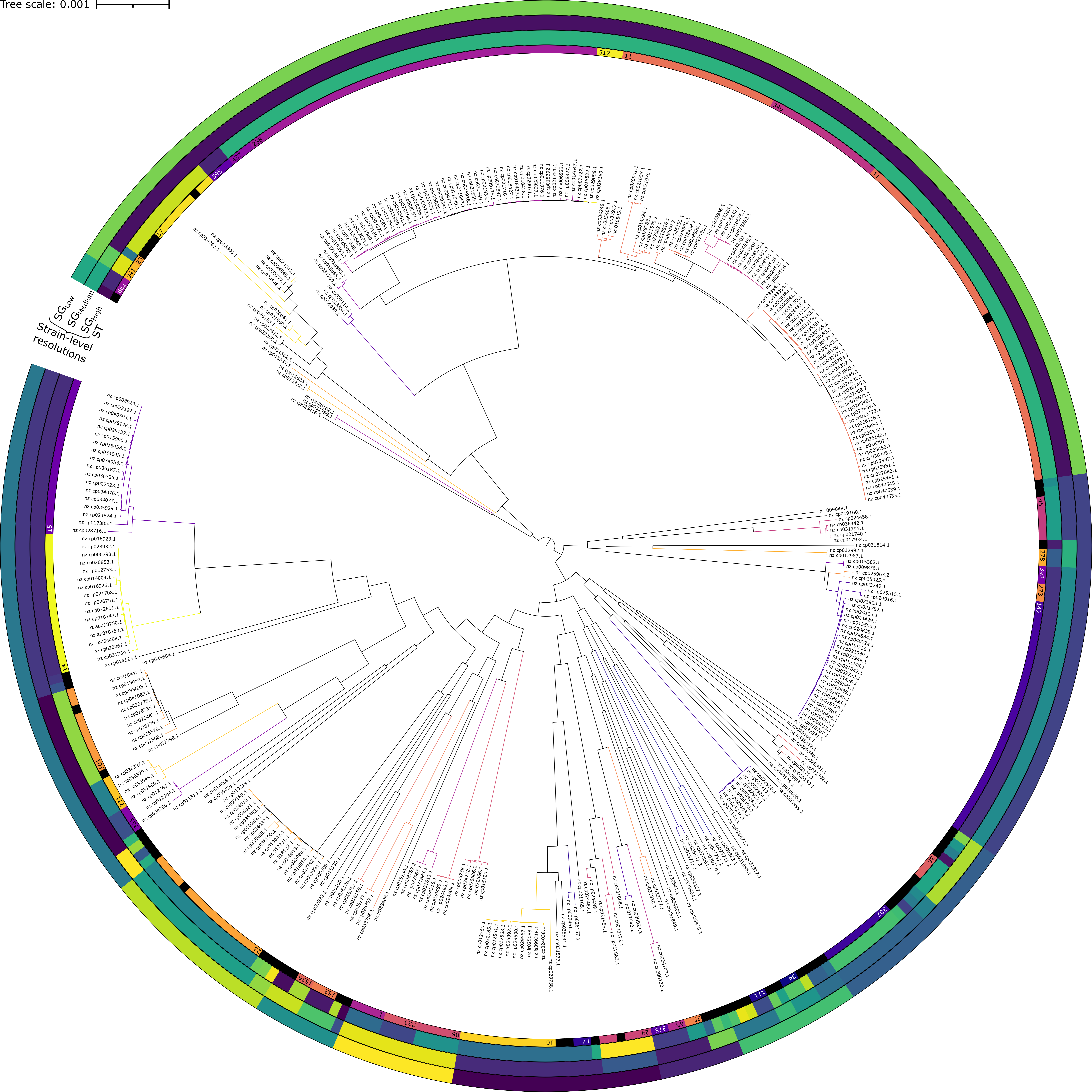

Supplement: Supplementary file 7 — Supplementary Figure 2 [file 43856_2023_259_MOESM7_ESM.pdf]
