# Supplementary material for: Strain-level bacterial typing directly from patient samples using optical DNA mapping
Source: Commun Med (Lond). 2023 Feb 23;3:31. doi: 10.1038/s43856-023-00259-z (PMC9950433; doi:10.1038/s43856-023-00259-z)
Supplement: Supplementary file 8 — Description of Additional Supplementary Files [file 43856_2023_259_MOESM8_ESM.pdf]

Description of Additional Supplementary Files

File Name: Supplementary Data 1

Description: A list of NCBI accession numbers of all sequences used to build the reference database.

File Name: Supplementary Data 2

Description: Lists of all *Escherichia coli/Shigella* spp. and *Klebsiella pneumoniae* genomes included in the reference database and their assigned strain group (SG) for the tested strain-level taxonomic resolutions: SG_{Low}, SG_{Medium}, SG_{High}, and, only for *E. coli*, $SG_{Ultra\text{-}High}$. For comparison to existing strain-level groupings, the lists also include type (ST) and, only for *E. coli*, their Clermont's phylogroup, for each reference sequence.

File Name: Supplementary Data 3

Description: A list of all the included STs for each SG at each of the tested strain-level resolutions.

File Name: Supplementary Data 4

Description: A list of all the analysed samples together with their species, sequence type, sequencing data accession number, references to relevant figures in the main text, and the numbers of experimental intensity profiles.

File Name: Supplementary Figure 1

Description: ***Escherichia coli* tree.** The phylogenetic tree of the Escherichia coli/Shigella spp. genomes in the reference database.

File Name: Supplementary Figure 2

Description: ***Klebsiella pneumoniae* tree.** The phylogenetic tree of the *Klebsiella pneumoniae* genomes in the reference database.
