# Supplementary material for: Strain-level bacterial typing directly from patient samples using optical DNA mapping
Source: Commun Med (Lond). 2023 Feb 23;3:31. doi: 10.1038/s43856-023-00259-z (PMC9950433; doi:10.1038/s43856-023-00259-z)
Supplement: Supplementary file 9 — Reporting Summary [file 43856_2023_259_MOESM9_ESM.pdf]

nature portfolio

Corresponding author(s): Fredrik Westerlund, Erik Kristiansson

Last updated by author(s): Jan 30, 2023

Reporting Summary

Nature Portfolio wishes to improve the reproducibility of the work that we publish. This form provides structure for consistency and transparency in reporting. For further information on Nature Portfolio policies, see our Editorial Policies and the Editorial Policy Checklist.

Statistics

For all statistical analyses, confirm that the following items are present in the figure legend, table legend, main text, or Methods section.

n/a	Confirmed	
☒	☐	The exact sample size (*n*) for each experimental group/condition, given as a discrete number and unit of measurement
☒	☐	A statement on whether measurements were taken from distinct samples or whether the same sample was measured repeatedly
☒	☐	The statistical test(s) used AND whether they are one- or two-sided<br>*Only common tests should be described solely by name; describe more complex techniques in the Methods section.*
☒	☐	A description of all covariates tested
☒	☐	A description of any assumptions or corrections, such as tests of normality and adjustment for multiple comparisons
☒	☐	A full description of the statistical parameters including central tendency (e.g. means) or other basic estimates (e.g. regression coefficient) AND variation (e.g. standard deviation) or associated estimates of uncertainty (e.g. confidence intervals)
☒	☐	For null hypothesis testing, the test statistic (e.g. *F*, *t*, *r*) with confidence intervals, effect sizes, degrees of freedom and *P* value noted<br>*Give P values as exact values whenever suitable.*
☒	☐	For Bayesian analysis, information on the choice of priors and Markov chain Monte Carlo settings
☒	☐	For hierarchical and complex designs, identification of the appropriate level for tests and full reporting of outcomes
☒	☐	Estimates of effect sizes (e.g. Cohen's *d*, Pearson's *r*), indicating how they were calculated

Our web collection on statistics for biologists contains articles on many of the points above.

Software and code

Policy information about availability of computer code

Data collection	For collection of fluorescence DNA images Carl Zeiss Zen Blue edition v2.6-3.4 were used.
Data analysis	The analysis of the DNA intensity profiles are MATLAB-based (using versions 2018b-2021b).<br>The code for consensus intensity profile analysis is contained in the lldev package and deposited at https://doi.org/10.5281/zenodo.5718208.<br>The code for generating intensity profiles and aligning them against in silico predicted intensity profiles is contained in the HCA package and deposited at https://doi.org/10.5281/zenodo.5718183.<br><br>The code for analysing the alignment results and identifying intensity profiles that match discriminatively to a single taxonomic group is deposited at https://doi.org/10.5281/zenodo.5898280.<br><br>The following tools were used for the construction of strain group schemes: Prokka version 1.12, Roary version 3.13.0, FastTree version 2.1.11, iTol version 6, R version 3.6.2, R package phytools version 0.6-99, R package dendextend version 1.13.4.<br>The following tools were used for assembly and annotation of the sequence samples: MLST version 2.0.4, EZClermont version 0.6.3, CLC Genomics Workbench version 21, Trim Galore! version 0.4.3, SPAdes version 3.14.1.

For manuscripts utilizing custom algorithms or software that are central to the research but not yet described in published literature, software must be made available to editors and reviewers. We strongly encourage code deposition in a community repository (e.g. GitHub). See the Nature Portfolio guidelines for submitting code & software for further information.

Data

Policy information about availability of data

All manuscripts must include a data availability statement. This statement should provide the following information, where applicable:

- Accession codes, unique identifiers, or web links for publicly available datasets
- A description of any restrictions on data availability
- For clinical datasets or third party data, please ensure that the statement adheres to our policy

Raw kymographs of individual molecules from the optical DNA mapping have been deposited with figshare and can be accessed at: https://doi.org/10.6084/m9.figshare.c.5760860.v1. A list of NCBI accession numbers of all sequences used to build the reference database is available as Supplementary Data 1. Supplementary Data 2 lists all Escherichia coli/Shigella spp. and Klebsiella pneumoniae genomes included in the reference database and their assigned strain group (SG) for the tested strain-level taxonomic resolutions: SGLow, SGMedium, SGHigh, and, only for E. coli, SGUltra-High. For comparison to existing strain-level groupings, the lists also include type (ST) and, only for E. coli, their Clermont's phylogroup, for each reference sequence. A list of all the included STs for each SG at each of the tested strain-level resolutions is available as Supplementary Data 3. A list of all the analysed samples together with their species, sequence type, sequencing data accession number, references to relevant figures in the main text, and the numbers of experimental intensity profiles are available as Supplementary Data 4. Raw sequencing data generated for this project have been submitted to NCBI SRA and all studied samples are linked to BioProject accession number PRJNA774113. All other data is available upon reasonable request from the corresponding author. Figure 2 (a-f), Figure 3 (a, b, d) and Figure 4b was generated using the data in Supplementary Data 4. Figure 3c was generated from the reference genomes listed in Supplementary Data 1 and the sequenced samples linked to the BioProject accession number PRJNA774113. Figure 4 (c, d) was generated using the kymographs for plasmids, where the kymographs and experimental settings (KymographInfo.xlsx) can be accessed at https://doi.org/10.6084/m9.figshare.c.5760860.v1.

Human research participants

Policy information about studies involving human research participants and Sex and Gender in Research.

Reporting on sex and gender	No human research participants were included in the study. Neither sex or gender is used throughout the study.
Population characteristics	No human research participants were included in the study.
Recruitment	No human research participants were recruited for the study.
Ethics oversight	No human research participants were included in the study.

Note that full information on the approval of the study protocol must also be provided in the manuscript.

Field-specific reporting

Please select the one below that is the best fit for your research. If you are not sure, read the appropriate sections before making your selection.

☒ Life sciences ☐ Behavioural & social sciences ☐ Ecological, evolutionary & environmental sciences

For a reference copy of the document with all sections, see nature.com/documents/nr-reporting-summary-flat.pdf

Life sciences study design

All studies must disclose on these points even when the disclosure is negative.

Sample size	The samples and sample size were determined from either clinical relevance or from their phylogenetic relationship for method evaluation.
Data exclusions	Experiments where the stretch factor were below 0.2 nm/bp for the reference molecules were excluded from further analysis as the resolution was deemed too poor.
Replication	Due to the method being single DNA based, and the DNA fragmentation is random when extracted, exact reproducibility is not expected.
Randomization	Not relevant for our study, since the method aims to identify bacteria at the strain level. The identity is confirmed by sequencing and no randomization is needed.
Blinding	Blinding is not relevant for our study, see randomization.

Reporting for specific materials, systems and methods

We require information from authors about some types of materials, experimental systems and methods used in many studies. Here, indicate whether each material, system or method listed is relevant to your study. If you are not sure if a list item applies to your research, read the appropriate section before selecting a response.

Materials & experimental systems

n/a	Involved in the study
☒	☐ Antibodies
☒	☐ Eukaryotic cell lines
☒	☐ Palaeontology and archaeology
☐	☒ Animals and other organisms
☒	☐ Clinical data
☒	☐ Dual use research of concern

Methods

n/a	Involved in the study
☒	☐ ChIP-seq
☒	☐ Flow cytometry
☒	☐ MRI-based neuroimaging

Animals and other research organisms

Policy information about studies involving animals; ARRIVE guidelines recommended for reporting animal research, and Sex and Gender in Research

Laboratory animals	The study did not involve any animals only bacteria E. coli and K. pneumoniae.
Wild animals	No wild animals in the study.
Reporting on sex	Not relevant since only E. coli and K. pneumoniae were used.
Field-collected samples	No field-collected samples were used.
Ethics oversight	No ethical approval was needed for the use of cultivated E. coli and K. pneumoniae.<br><br>No informed consent was collected from patients with urinary tract infections, as per the Swedish Ethical Review Authority assessment (recordal 2018/273531/2). We included only samples that were ready to be discarded and no additional sampling from patients was needed.

Note that full information on the approval of the study protocol must also be provided in the manuscript.
